# Multi and serial data collection and processing

**DOI:** 10.1107/S2059798319002870

**Published:** 2019-02-26

**Authors:** Robin Owen, Max Nanao, Arwen R Pearson

**Affiliations:** a Diamond Light Source, Harwell Science and Innovation Campus, Didcot OX11 0DE, UK; b Structural Biology Group, European Synchrotron Radiation Facility, 71 Avenue des Martyrs, 38000 Grenoble, France; c Institute for Nanostructure and Solid State Physics, Unversität Hamburg, CFEL, Bldg. 99, Luruper Chaussee 149, Hamburg, 22761, Germany

**Keywords:** CCP4 Study Weekend, Multi and serial data collection and processing

## Abstract

An introduction to the 2018 CCP4 Study Weekend special issue.

The 2018 CCP4 Study Weekend was held at the University of Nottingham with the title *Multi and serial data collection and processing*. The meeting ranged from why we might need to consider multi-crystal data collection, though the software and hardware making it possible, to how we can best process and interpret the data obtained.

The first session started with James Holton reminding us that multi-crystal data collection is nothing new and in fact pre-dates single crystal data collections. For example, 22 precession photographs were used by Kendrew *et al.* to solve myoglobin in the early 1960s. He went on to note that non-isomorphism is one of the biggest challenges in multi-crystal techniques, and demonstrated, as an example, how even minor changes in monomer positioning (rotations) or unit cells can lead to large merging *R* values between crystals. In this issue, a ‘challenge data set’ is presented and made available to developers of new multi-crystal algorithms. Daniel Keedy followed, with a demonstration of how some conformational modes may be hidden at cryogenic temperatures necessitating the need for elevated temperature data collection (and more crystals). In this issue he discusses how the equilibrium between these hidden modes can be shifted by ligand binding and/or temperature jumps in order to reveal their structures.

Kunio Hirata described the *ZOO* system of beamline tools for collecting data from many crystals in an efficient manner. *ZOO*, described in detail in this issue, can handle different modes of data collection, including full single crystal sweeps, mini oscillations and single-image data collections. Some of these modes can even be mixed on a single sample holder (the *HITO* sub-system).

Mike Hough closed the session by explaining how many structures can be obtained from a single or very limited number of crystals by taking advantage of the combination of fast detectors and shutterless data collection to record successive low-dose data sets from a crystal to provide snapshots along a reaction pathway. In this issue he presents the use of a fixed-target approach serial crystallographic experiment that could both resolve multi polymorphs present in the microcrystal population and provide very low dose data collection.

The second session focused on sample preparation and getting samples into the X-ray beam. Aina Cohen discussed developments at SLAC and the LCLS in sample positioning, sample harvesting, and a new beamline under construction with serial capabilities, while Gabriella Nass Kovacs provided an excellent overview of viscous matrix injection technologies. They have summarized their talks in two complementary articles in this issue. Thomas Schneider completed the session by taking us through different approaches for serial delivery of crystals at X-ray free-electron lasers and synchrotrons, and discussing how the goal of the experiment defines the optimal delivery method. In this issue he discusses the *Mesh&Collect* data collection approach for long-wavelength SAD phasing using microcrystals. Particularly exciting in this session was seeing the cross-fertilization between FEL and synchrotron in terms of sample delivery for serial FEL and synchrotron experiments.

The final session of the day moved onto how to process multi-crystal and serial data. The session was opened by Karol Nass who presented a two-colour XFEL experiment, to study radiation damage in FEL experiments, and discussed the challenges that arose during the data processing. In this issue he presents a comprehensive review of radiation damage during FEL experiments. Graeme Winter discussed the advantages of having weak data from many crystals rather than strong data from a few and gave some pointers on how to make the best use of available photons. Helen Ginn and Thomas White then talked about the challenges in defining and refining the geometry of an experiment when each crystal provides only a single still, and often weak, diffraction image. In this issue Thomas White has provided a detailed, step-by-step guide to using *CrystFEL* to process serial crystallographic data.

The first session of the second day was opened by Kay Diederichs who spoke about the challenges in merging many incomplete data sets that may or may not be truly isomorphous, and discussed a new method for separating isomorphous from non-isomorphous error. Richard Gildea and Mona Uervirojjnangkoorn continued on this theme, covering symmetry determination from partial data sets and resolution of indexing ambiguities. Richard discussed an extension of the Brehm and Diederichs method for resolving indexing ambig­uity called Cosym, which is particularly useful for partial data sets. In this issue Mona Uervirojjnangkoorn presents another extension of the Brehm and Diederichs method to include pseudomerohedral indexing ambiguities and demonstrates its application to an XFEL data set for the SNARE–complexin-1–synaptotagmin-1 complex. Gianluca Santoni closed the session on a different note: the use of cluster analysis to identify optimal combinations of data sets.

The second session of the day moved onto phasing, refinement and validation. George Sheldrick covered how *SHELX* can be used to merge and phase multi-crystal data and detailed some metrics for success. Nicolas Foos described the use of a genetic algorithm in efficiently forming an isomorphous complete data set from potentially non-isomorphous subsets and provides further detail of this approach in this issue. Vincent Olieric gave several examples of optimized data collection strategies for successful sulfur SAD, and discussed some of the challenges involved, such as sample and air absorption at longer wavelengths.

The final session opened with a talk from Xiaodong Zou on collection of electron diffraction data from multiple crystals and compared it with X-ray diffraction, noting in particular the stronger interaction of electrons with atoms, as well as much lower damage per scattering event, but also the typically higher *R* values. She additionally described an automated procedure for electron diffraction that she has developed called RED (Rotation Electron Diffraction). This method has also been used for multiple crystals to determine the structure of a zeolite. The final talk of the meeting from Janet Smith looked towards the future, discussing the future of X-ray crystallography at synchrotrons with micro- and nano-beams and the recent pink beam serial crystallography experiments at the Laue beamline at the APS, as well as injector experiments.

We would like to thank Karen McIntyre and Charles Ballard for their assistance in organizing the 2018 Study Weekend, the speakers for their stimulating talks and the session chairs for keeping the meeting running to time!

